# Baicalein reduces β-amyloid and promotes nonamyloidogenic amyloid precursor protein processing in an Alzheimer’s disease transgenic mouse model

**DOI:** 10.1002/jnr.23244

**Published:** 2013-05-17

**Authors:** She-Qing Zhang, Demian Obregon, Jared Ehrhart, Juan Deng, Jun Tian, Huayan Hou, Brian Giunta, Darrell Sawmiller, Jun Tan

**Affiliations:** 1 Department of Neurology, Shanghai Changhai HospitalShanghai, China; 2 Rashid Laboratory for Developmental Neurobiology Silver Child Development Center Department of Psychiatry and Behavioral Neurosciences, Morsani College of Medicine, University of South FloridaTampa, Florida; 3 Neuroimmunology Laboratory Department of Psychiatry and Behavioral Neurosciences, Morsani College of Medicine, University of South FloridaTampa, Florida

**Keywords:** β-amyloid, Alzheimer’s disease, flavonoid, baicalein, sAPPα, GABA

## Abstract

Baicalein, a flavonoid isolated from the roots of *Scutellaria baicalensis*, is known to modulate γ-aminobutyric acid (GABA) type A receptors. Given prior reports demonstrating benefits of GABA_A_ modulation for Alzheimer’s disease (AD) treatment, we wished to determine whether this agent might be beneficial for AD. CHO cells engineered to overexpress wild-type amyloid precursor protein (APP), primary culture neuronal cells from AD mice (Tg2576) and AD mice were treated with baicalein. In the cell cultures, baicalein significantly reduced the production of β-amyloid (Aβ) by increasing APP α-processing. These effects were blocked by the GABA_A_ antagonist bicuculline. Likewise, AD mice treated daily with i.p. baicalein for 8 weeks showed enhanced APP α-secretase processing, reduced Aβ production, and reduced AD-like pathology together with improved cognitive performance. Our findings suggest that baicalein promotes nonamyloidogenic processing of APP, thereby reducing Aβ production and improving cognitive performance, by activating GABA_A_ receptors. © 2013 Wiley Periodicals, Inc.

Alzheimer’s disease (AD) is a neurodegenerative disease characterized pathologically by senile plaques (SPs) and neurofibrillary tangles (NFTs) and clinically by progressive cognitive deterioration. Over the last 2 decades, the β-amyloid (Aβ) hypothesis has been the dominant theory for the mechanism of AD pathogenesis (Hardy and Higgins, 1992; Hardy et al., 1998; Hardy and Selkoe, 2002). Amyloid precursor protein (APP) proteolysis is the fundamental process for the production of Aβ peptides implicated in AD pathology. Aβ peptides are produced by the initial action of β-secretase (BACE) cleavage, which creates an Aβ-containing C-terminal fragment (CTF) known as β-CTF and an N-terminal, soluble APP-β (sAPPβ) fragment, which is released extracellularly. Intracellularly, β-CTF is then cleaved by a multiprotein γ-secretase complex that results in generation of the Aβ peptide and a smaller γ-CTF. Under physiological conditions, Aβ is constitutively generated at relatively low levels and, hence, most APP is processed by the nonamyloidogenic pathway involving α-secretase. In the nonamyloidogenic pathway, APP is first cleaved at the α-secretase site, which results in the release of N-terminal sAPPα and the generation of α-CTF. Because of the limiting amount of APP in the cell and the failure to saturate the BACE pathway during APP overexpression, it is believed that the above-mentioned amyloidogenic and nonamyloidogenic pathways compete for substrate in the process of APP proteolysis (Hardy and Selkoe, 2002). Therefore, it is often inferred that extracellular elevation of sAPPα can be taken as indirect evidence of activation of α-secretase or inhibition of BACE and the associated amyloidogenic pathway (Rezai-Zadeh et al., 2005).

Over the past decade, intense focus has been given to investigating the processes of APP proteolysis and Aβ metabolism as possible targets for AD therapy. One naturally occurring compound is baicalein (5,6,7-trihydroxyflavone), extracted from the root of the Chinese medicinal herb *Scutellaria baicalensis* Georgi (Jianjun and Huiru, 2008). Previous studies have demonstrated the usefulness of baicalein in reducing cognitive deficits in rats induced by chronic cerebral hypoperfusion and improving memory in ibotenic acid-induced rat models (Liu et al., 2007; Heo et al., 2009). Further, Wang et al. (2004) have shown baicalein can reduce β-amyloid peptide-(25–35)-induced amnesia in mice. Heo et al. (2004) reported that baicalein potently reduces Aβ-induced neurotoxicity in PC12 cells, possibly by a reduction of oxidative stress. Likewise, Zhu et al. (2004) observed that baicalein can inhibit fibrillation of α-synuclein and disaggregate existing fibrils, which raises the possibility of baicalein as a potential treatment for neurodegenerative diseases, such as Parkinson’s disease. However, whether baicalein can directly inhibit Aβ production and ameliorate the subsequent cognitive impairments observed in an animal model of AD has not been determined.

The present study shows that baicalein significantly decreased Aβ production both in Chinese hamster ovary cells expressing a human wild-type APP gene (CHO/APPwt) and in primary neurons from transgenic Tg2576 mice. In concert with these observations, we found that baicalein promotes cleavage of α-CTF and elevates sAPPα. These cleavage events are attenuated with a specific GABA receptor antagonist, bicuculline. As a validation of these findings in vivo, we treated Aβ-overproducing Tg2576 transgenic mice with baicalein for 8 weeks and found decreased Aβ levels in the brain associated with promotion of the nonamyloidogenic α-secretase proteolytic pathway. Furthermore, we found the cognitive performance of these mice to be significantly improved compared with untreated mice, suggesting that this natural compound should be tested further for translation into humans.

## MATERIALS AND METHODS

### Reagents and Antibodies

Baicalein powder (99% purity by high-pressure liquid chormatography) was purchased from Shanghai Jiahe (Shanghai, China). The reagent was initially dissolved in dimethylsulfoxide (DMSO) and then diluted in PBS (pH 7.4) to a final concentration of 0–10 μM and less than 1% DMSO (de Carvalho et al., 2011). Bicuculline methiodide (BMI) was purchased from Sigma-Aldrich Chemie (Munich, Germany), prepared initially in distilled water and diluted in PBS to a final concentration of 10 μM (Behrens et al., 2007).

Sterile, azide-free, and low-endotoxin antibodies were used, including anti-C-terminal human sAPPα-specific antibody (2B3; 100 μg/ml; IBL, Minneapolis, MN), Aβ_1–17_ antibody (6E10; 1 mg/ml; Covance, Emeryville, CA), APP C-terminal antibody (pAb751/770; 500 μg/ml; EMD Biosciences, La Jolla, CA), and β-actin antibody (100 μg/ml; Sigma-Aldrich). The sAPPα-specific 2B3 antibody was further characterized in our in vitro and in vivo systems, indicating that this antibody recognizes neither Aβ nor full-length APP.

### Cell Culture

CHO cells engineered to express wild-type human APP (CHO/APPwt) were kindly provided by Dr. Stefanie Hahn and Dr. Sascha Weggen, (University of Heinrich Heine, Düsseldorf, Germany). These cells were maintained in Dulbecco’s modified Eagle’s medium with 10% bovine serum, 1 mM sodium pyruvate, and 100 U/ml penicillin/streptomycin, as described previously (Hahn et al., 2011). Tg2576 mouse-derived neuronal cells were cultured as described previously (Obregon et al., 2006). Briefly, cerebral cortices isolated from 1-day-old Tg2576 mice were mechanically dissociated in trypsin (0.25%) individually after incubation for 15 min at 37°C. Cells were collected after centrifugation at 1,200*g*, suspended in Dulbecco’s modified Eagle’s medium supplemented with 10% fetal calf serum, 10% horse serum, uridine (33.6 μg/ml; Sigma), and fluorodeoxyuridine (13.6 μg/ml; Sigma) and seeded in 24-well collagen-coated culture plates at 2.5 × 10^5^ cells per well. After reaching confluence (approximately 70–80%), cells were treated with baicalein at 0–10 μM for 12 hr. In some experiments, cells were treated with baicalein at 5 μM in the absence or presence of bicuculline at 10 μM.

### Mice

Transgenic mice overexpressing “Swedish” mutant APP (695-aa isoform) under control of the prion promoter (line 2576, Tg2576) were purchased from Taconic Farms (Germantown, NY). For i.p. administration of baicalein, 12 female Tg2576 mice were used; seven mice received baicalein, and five control mice received PBS. Beginning at 6 months of age, these mice were injected i.p. with baicalein (10 mg/kg) or PBS daily for 8 weeks (Rezai-Zadeh et al., 2005). In addition, similarly aged nontransgenic (NT) mice were concurrently given daily i.p. injections of baicalein (10 mg/kg) or PBS for 8 weeks. All mice were sacrificed at 8 months of age for analysis of Aβ levels and APP processing products in brain homogenates according to previously described methods (Rezai-Zadeh et al., 2005; Zhu et al., 2011). All behavioral testing occurred during the final weeks preceding sacrifice, with treatment being continued. The mice were housed and maintained at the University of South Florida (USF) and all experiments were in compliance with protocols approved by the USF Institutional Animal Care and Use Committee.

### Tissue Preparation

Mice were euthanized with isoflurane and transcardially perfused with ice-cold phosphate-buffered saline (PBS). Brains were isolated under sterile conditions on ice, placed in ice-cold lysis buffer (20 mM Tris, pH 7.5, 150 mM NaCl, 1 mM EDTA, 1 mM EGTA, 1% v/v Triton X-100, 2.5 mM sodium pyrophosphate, 1 mM β-glycerolphosphate, 1 mM Na_3_VO_4_, 1 μg/ml leupeptin, 1 mM PMSF), sonicated for approximately 3 min, allowed to stand for 15 min at 4°C, and then centrifuged at 15,000 rpm for 15 min. Supernatants were collected for analyses of soluble Aβ levels by ELISA and Western blot (WB) analysis, according to methods described previously (Rezai-Zadeh et al., 2005). The pellet was extracted with 5 M guanidine for analysis of insoluble Aβ as described previously (Zhu et al., 2011).

### Western Blot Analyses

Cells were washed with ice-cold PBS three times, lysed with cell lysis buffer (Cell Signaling Technology, Danvers, MA), and stored at −80°C until use. Aβ species secreted from cells and in brain homogenates were analyzed by Aβ ELISA and WB (Zhu et al., 2011). Briefly, for WB analysis, 10% bicine/tris gel containing 8 M urea was used for separation of proteins from brain homogenates or CHO conditioned media. Proteins were then transferred to 0.2-μm-pore-size nitrocellulose membranes (Bio-Rad, Hercules, CA). The membrane was boiled in PBS for 3–5 min before blocking to enhance sensitivity. 2B3 antibody was used for detection of sAPPα, and 6E10 was used for detection of Aβ. The 16.5% Tris tricine gel was used for detection of APP CTFs using pAB751/770. Densitometry analysis was performed with a FluorS Multimager with Quantity One software.

### ELISA

Aβ_40,42_ species in cell conditioned media and brain homogenates were detected by Aβ_40,42_ ELISA kits (Invitrogen, Carlsbad, CA). The manufacturer’s instructions were strictly followed. The Aβ_40,42_ levels are represented as picograms of Aβ_40_ or Aβ_42_ per milliliter of conditioned media or as picograms of Aβ_40_ or Aβ_42_ per milligram of total cellular protein.

### Radial Arm Water Maze

The radial arm water maze (RAWM) test was conducted according to that previously described by Arendash et al. (2001). Briefly, the maze is composed of a 100-cm circular pool with six swim alleys (30.5 cm long and 19 cm wide) radiating from a common circular swim area 40 cm in diameter. An assortment of spatial cues is located on the testing room’s walls and ceiling. A submerged escape platform 9 cm in diameter is placed near the end of a different arm for each day of testing, which forces mice to use working memory to perform this task. A semirandom sequence of the remaining five arms is then selected as start arms for each of that day’s trials. A different sequence of start arms is selected each day, which never includes that day’s goal arm. Four consecutive acquisition trials (T1–T4) give animals the opportunity to learn which arm contains the submerged platform for that day. A retention trial (T5), beginning from the one remaining start arm for that day, is administered 30 min after T4.

For any given trial, animals are placed in the designated start arm facing the common circular swim area. During the following 1-min maximum trial, each time an animal enters an incorrect arm (or fails to select an arm after 20 sec), it is gently pulled back to the start arm for that trial, and an error is charged. For each trial, the number of errors prior to escape onto the goal arm’s submerged platform is recorded, as is the latency to escape. If the submerged platform is not located within a given 1-min trial, the mouse is guided to the submerged platform and assigned a latency score of 60 sec. Animals are allowed to remain on the platform for 30 sec before the next trial begins. Mice were tested after 8 weeks of baicalein or PBS treatment. Two animals in the baicalein treatment group were eliminated from the final data analysis because of a consistent lack of arm choices throughout the test period.

### Statistical Analysis

All data were normally distributed. In instances of single mean comparisons, Levene’s test for equality of variances followed by *t*-test for independent samples was used to assess significance. In instances of multiple mean comparisons, analysis of variance was used, followed by post hoc comparison using Bonferonni’s method. Alpha levels were set at 0.05 for all analyses. The statistical package for the social sciences release 10.0.5 (SPSS Inc., Chicago, IL) was used for all data analysis.

## RESULTS

### Baicalein Inhibits Aβ_40,42_ Generation From CHO/APPwt Cells and Tg2576 Mouse-Derived Neurons

**Fig 1:**
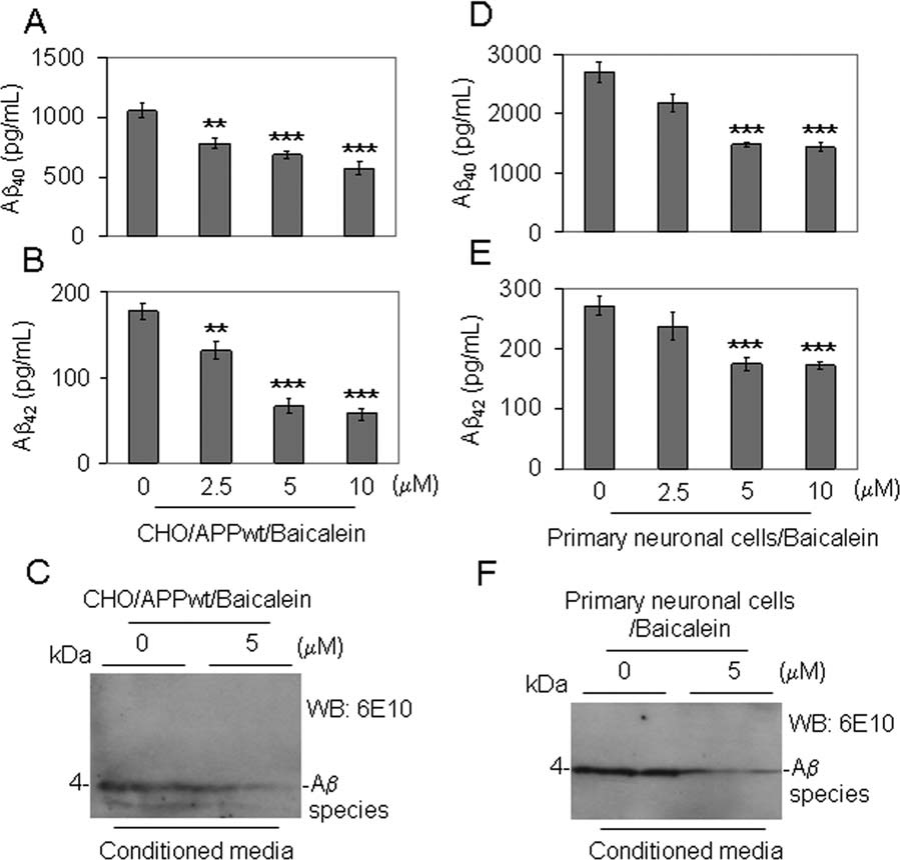
Baicalein inhibits Aβ generation in cultured cells. CHO cells expressing wild-type human APP (CHO/APPwt) and primary neuronal cells cultured from brain tissues of 1-day-old Tg2576 mice were treated with baicalein at 0, 2.5, 5, and 10 μM as indicated for 12 hr, followed by analysis of Aβ_40,42_ peptides secreted in the cell culture media by Aβ ELISA (A,B,D,E) and IB analysis (C,F). The Aβ ELISA results are represented as mean ± SD of Aβ_40_ or Aβ_42_ (pg/ml) in cell culture media after baicalein treatment. These results are representative of three independent experiments, with n = 3 for each condition. One-way ANOVA followed by post hoc comparison revealed significant differences between 2.5, 5, or 10 μM and 0 μM baicalein treatment in Aβ_40,42_ reduction. **P < 0.01, ***P < 0.005.

To examine the effects of baicalein on Aβ production, we first treated CHO/APPwt cells and primary neurons derived from Tg2576 mice with a wide dose range of baicalein for 12 hr and then measured Aβ_40_ and Aβ_42_ generation by ELISA. Baicalein reduced both Aβ_40_ and Aβ_42_ generation in CHO/APPwt cells (Fig. 1A–C) and primary Tg2576-derived neurons in a dose-dependent manner (Fig. 1D–F). Baicalein at 10 μM reduced Aβ_40_ and Aβ_42_ generation from CHO/APPwt cells by 49% and 66% (Fig. 1A,B), respectively, and reduced Aβ_40_ and Aβ_42_ generation from primary Tg2576-derived neurons by 50% and 37% (Fig. 1D,E), respectively, compared with untreated cells. To address this result further, we treated CHO/APPwt cells and Tg2576-derived neurons with 5 μM baicalein and determined Aβ_40_ and Aβ_42_ secreted into the cell media by WB analysis, using 6E10 antibody. It was noted that Aβ secretion into the cell media was significantly reduced for both CHO/APPwt and primary Tg2576-derived neurons (Fig. 1C,F).

### Baicalein Activates Nonamyloidogenic Processing of APP in CHO/APPwt Cells

**Fig 2:**
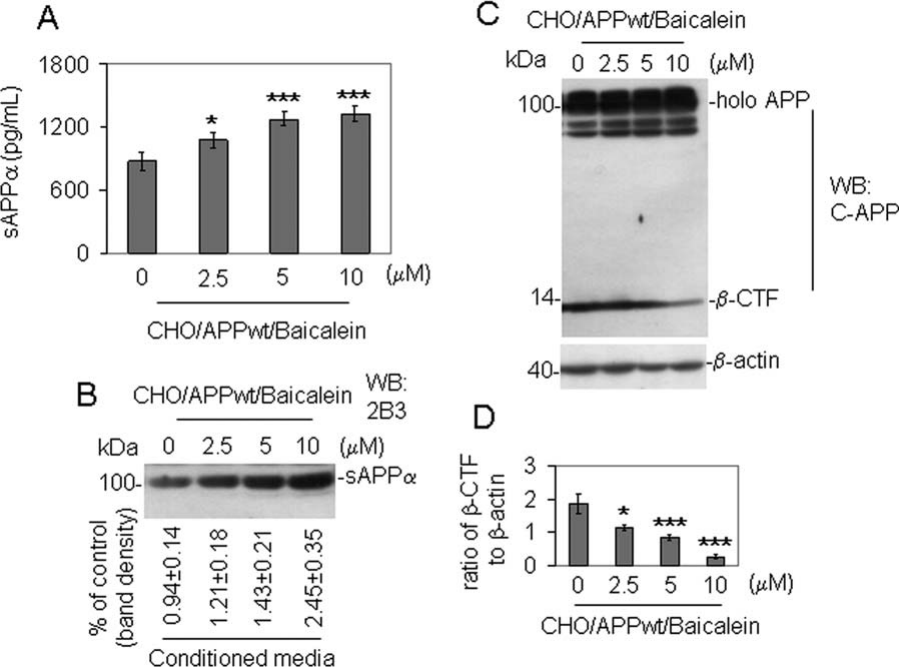
Baicalein increases nonamyloidogenic APP cleavage in cultured cells. CHO/APPwt cells were treated with baicalein at 0, 2.5, 5, and 10 μM as indicated for 12 hr. Secreted sAPPα in the cell culture media were analyzed by sAPPα ELISA (A) and WB (B), using 2B3 antibody. The sAPPα ELISA results are represented as the mean ± SD of sAPPα (pg/ml) in the cell culture media after baicalein treatment. Relative band density over vehicle control (1% DMSO in PBS; mean ± SD) was calculated by densitometry analysis as shown below the WB panel. These results are representative of three independent experiments, with n = 3 for each condition. C: Cell lysates were prepared, and APP CTFs were analyzed by WB using a rabbit polyclonal antibody against C-terminal APP (pAb751/770, C-APP). This β-CTF band was further confirmed by the additional WB using 6E10 antibody (data not shown). D: Relative ratio (mean ± SD) of β-CTF to β-actin was calculated by densitometry analysis. The results are representative of three independent experiments, with n = 3 for each condition. One-way ANOVA followed by post hoc comparison revealed significant differences between 2.5, 5, or 10 μM and 0 μM baicalein treatment in both the increased sAPPα and the decreased relative ratio of β-CTF to β-actin. *P < 0.05, ***P < 0.001, full-length APP, holo APP.

To elucidate the mode of action of baicalein on APP cleavage, we examined APP cleavage profiles after treatment of CHO/APPwt cells with baicalein using ELISA and WB analyses. At first, we treated CHO/APPwt cells with different dosages of baicalein for 12 hr and the secretion of sAPPα into the cell culture media was analyzed by ELISA and WB, using 2B3 antibody. In concert with decreased Aβ generation, baicalein increased sAPPα in a dose-dependent manner (Fig. 2A,B), suggesting that α-secretase activity was promoted. To confirm this result, we further analyzed the APP C-terminal fragments (CTF) in the cell lysates by WB using a rabbit polyclonal antibody against C-terminal APP (pAb751/770, C-APP). We noted that baicalein treatment significantly reduced the production of β-CTF in the CHO/APPwt cell lysates, indicative of inhibition of the amyloidogenic pathway (Fig. 2C,D). This was confirmed by additional WB for β-CTF using 6E10 antibody (data not shown).

### GABA_A_ Receptor Antagonist Bicuculline Blocks Baicalein-Mediated Aβ Reduction

**Fig 3:**
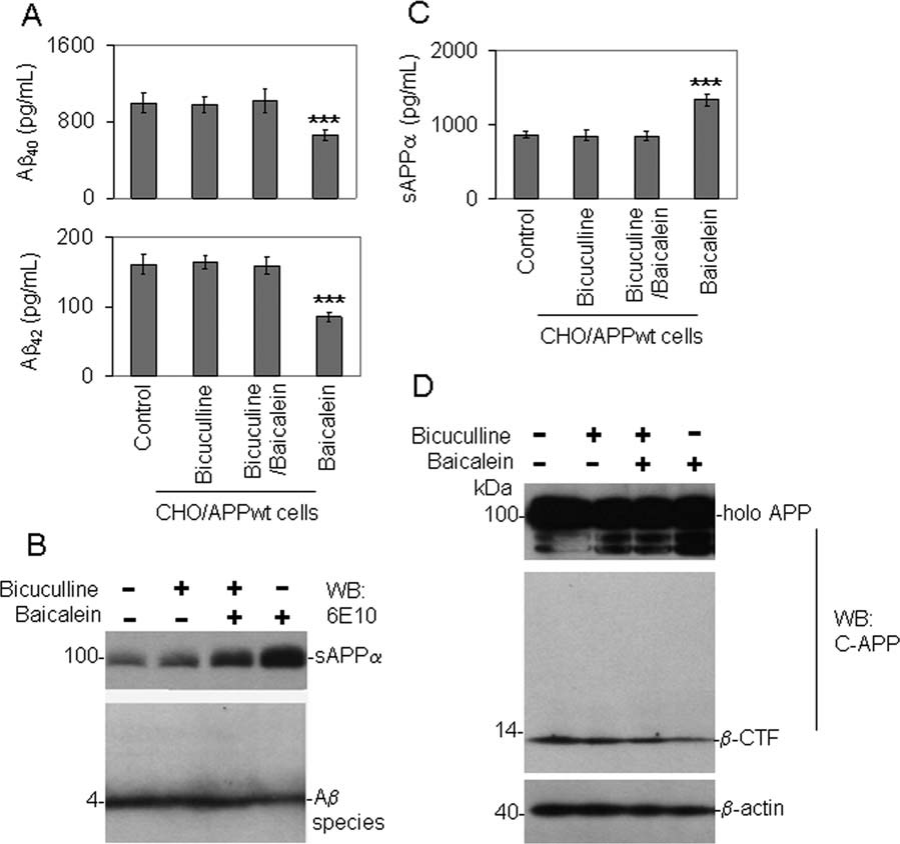
GABA antagonist blocks baicalein-mediated Aβ reduction. CHO/APPwt cells were treated with baicalein at 5 μM in the presence of GABA antagonist (bicuculline, 10 μM) for 12 hr. Secreted Aβ_40,42_ peptide (A,B) and sAPPα (B,C) were analyzed in the cell culture media by ELISA and WB analysis using 6E10 antibody. These results are representative of three independent experiments, with n = 3 for each condition. D: Cell lysates were prepared, and APP CTFs were analyzed by WB using a rabbit polyclonal antibody against C-terminal APP (pAb751/770; C-APP). This β-CTF band was further confirmed by the additional WB using 6E10 antibody (data not shown). Notably, bicuculline markedly abolished baicalein-mediated anti-Aβ- and α-secretase-promoting effects. ***P < 0.005.

A previous study by de Carvalho et al. (2011) has documented that the sedative effect of baicalein is associated with activation of the nonbenzodiazepine binding site at GABA_A_ receptors. To investigate the mechanism by which baicalein reduces Aβ production, we treated CHO/APPwt cells with 5 μM baicalein in the absence or presence of 10 μM bicuculline, a selective GABA_A_ receptor antagonist. Secreted Aβ_40,42_ peptides and sAPPα in the cell culture media were determined by ELISA and WB analyses. Bicuculline blocked baicalein-mediated reduction of Aβ (Fig. 3A,B), enhancement of sAPPα (Fig. 3B,C) and reduction of β-CTF (Fig. 3D). However, neither Aβ, nor sAPPα, nor β-CTF was altered by bicuculline alone (Fig. 3A–D). GABA_A_ receptor expression was detected in cell lysates prepared from cultured CHO/APPwt cells by WB analysis using a rabbit antibody against GABA_A_ receptor (06–868; EMD Millipore, Billerica, MA).

### Baicalein Promotes Nonamyloidogenic APP Processing and Reduces Aβ Levels in Tg2576 Mice

**Fig 4:**
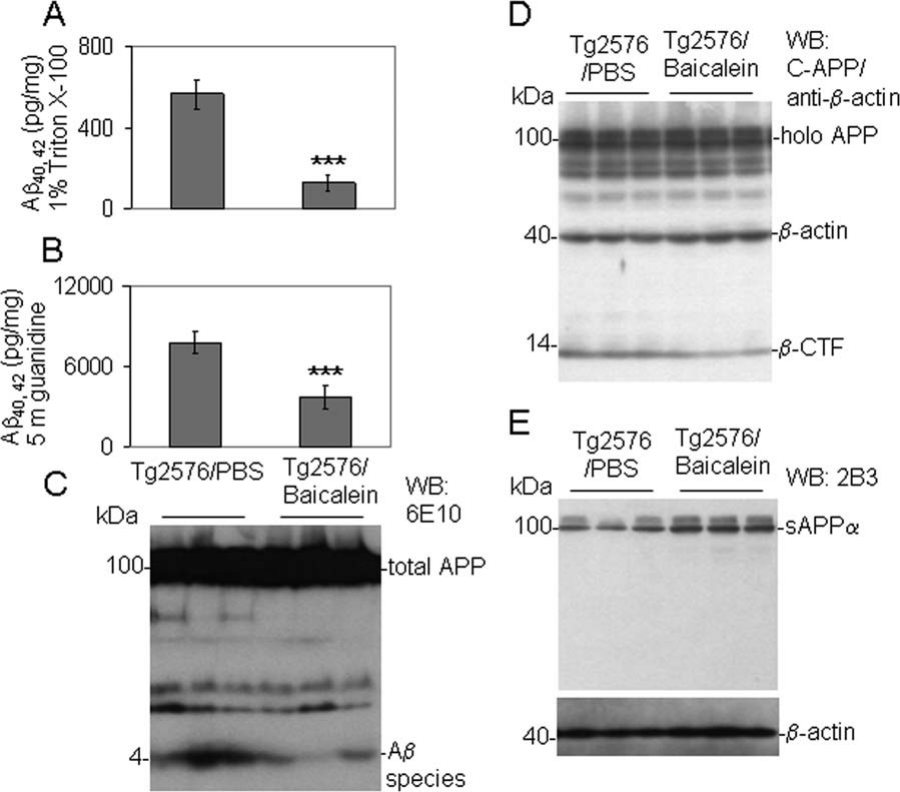
Baicalein promotes nonamyloidogenic APP processing and reduces cerebral Aβ levels in Tg2576 mice. Mouse brain homogenates were prepared from Tg2576 mice treated with baicalein or PBS for analysis of detergent-soluble (A) and -insoluble (B) Aβ_40,42_ prepared with 5 M guanidine by ELISA. In addition, soluble Aβ species were analyzed by WB using 6E10 (C). Data are presented as mean ± SD of Aβ_40,42_ (pg/mg protein) in brain homogenates. For A and B, a *t*-test revealed a significant between-groups difference for either soluble or insoluble Aβ_40,42_. ***P < 0.01. Brain homogenates were also prepared for analysis of β-CTF by pAb751/770 antibody (D) and sAPPα by 2B3 antibody (E).

We also investigated whether baicalein treatment could promote nonamyloidogenic APP processing and thereby impact cerebral Aβ levels in AD transgenic mice. Baicalein (10 mg/kg; n = 5) based on our previous study (Rezai-Zadeh et al., 2005) or PBS (n = 5) was injected i.p. daily for 8 weeks into 6-month-old female Tg2576 mice, and Aβ levels were determined by ELISA and WB. Aβ levels were markedly decreased in brain homogenate of Tg2576 mice treated with baicalein compared with those treated with PBS (Fig. 4A–C). As shown, Triton X-100 detergent-soluble Aβ_40,42_ and detergent-insoluble Aβ_40,42_ prepared with guanidine were reduced by 71%, and 53% respectively, compared with PBS-treated mice, as determined by ELISA (Fig. 4A,B). This baicalein-mediated reduction of soluble Aβ_40,42_ species was confirmed by WB using 6E10 antibody (Fig. 4C). In concert with the Aβ reduction, baicalein significantly reduced β-CTF (Fig. 4D) and significantly increased sAPPα in brain homogenate of Tg2576 mice (Fig. 4E) compared with PBS controls.

### Baicalein Treatment Improves Cognitive Performance of Tg2576 Mice

**Fig 5:**
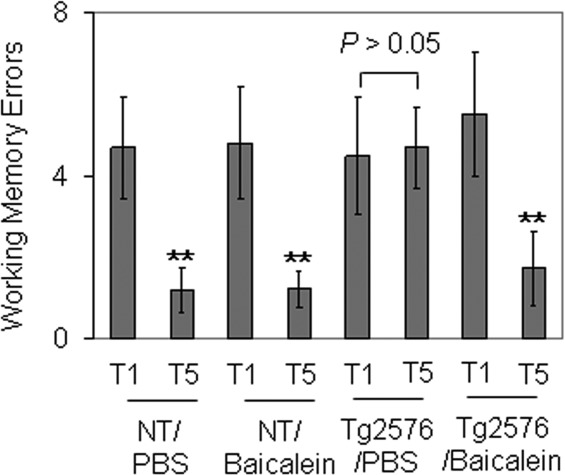
Baicalein treatment significantly improves cognitive performance of Tg2576 mice. During the final weeks of 8-week treatment, all four groups of mice went to behavioral testing. PBS-injected Tg2576 mice were significantly impaired compared with both PBS- and baicalein-injected NT control mice, as determined by trial 5 (T5) working memory errors. Baicalein-treated Tg2576 mice performed significantly better than PBS-injected Tg2576 mice (*P* < 0.05) and no differently from either PBS- or baicalein-injected NT control mice. Most interestingly, baicalein-treated Tg2576 mice and PBS- or baicalein-injected NT control mice, but not PBS-injected Tg2576 mice, were able to decrease their working memory errors significantly in trial 5 (T5) compared with trial 1 (T1). **P < 0.01. PBS-injected Tg2576 mice made substantially more T5 errors compared with the other three groups.

Inasmuch as baicalein reduced APP amyloidogenic processing in Tg2576 mice, we speculated that baicalein would improve cognitive performance in these mice as well. To test our speculation, we determined working memory in the baicalein- or PBS-treated Tg2576 mice, as well as NT control mice, by the RAWM method. We found that the cognitive performance of the PBS-injected Tg2576 mice was significantly impaired compared with baicalein- or PBS-treated NT control mice in trial 5 (T5) working memory errors (Fig. 5). In addition, baicalein-treated Tg2576 mice performed significantly better than PBS-injected Tg2576 mice but no different from both baicalein- or PBS-treated NT control mice. Most interestingly, baicalein-treated Tg2576 mice and baicalein- or PBS-treated NT control mice, but not PBS-treated Tg2576 mice, were able to decrease their working memory errors significantly in T5 compared with T1 (Fig. 5).

## DISCUSSION

Since the proposition of the Aβ hypothesis, many therapeutic studies on AD have focused on clinical trials of potential treatments that can reduce the production of Aβ; however, to date, there is no safe and effective agent that can be used clinically (Panza et al., 2010). Our present study demonstrates for the first time that baicalein can directly reduce the production of Aβ from CHO/APPwt cells in a dose-dependent manner (Fig. 1A–C). This result was seen not only in CHO/APPwt cells but also in primary neuron cells cultured from Tg2576 mice (Fig. 1D–F). This finding was associated with the elevation of sAPPα production in the cell media and reduced β-CTF production in the cell lysate, indicating that baicalein promoted nonamyloidgenic APP processing in these cells (Fig. 2A–D). In concert with these findings, we found that baicalein can significantly reduce the Aβ_40,42_ levels in the brains of an AD mouse model (Tg2576; Fig. 4A–C). These effects were associated with the promotion of sAPPα and reduction of β-CTF, indicating promotion of nonamyloidgenic APP processing (Fig. 4D,E). As was anticipated, the working memory errors tested in RAWM were also decreased after 8 weeks of baicalein treatment (Fig. 5). This indicated that baicalein can improve the learning and spatial memory abilities of Tg2576 mice.

These findings are very important in evaluating the potential use of baicalein in AD treatment as sAPPα is a known neurotrophic and neuroprotective factor. Previous studies have indicated that sAPPα can stimulate long-term potentiation, which is critical for learning and memory, and can also promote the outgrowth of neuritis, synaptogenesis, and synaptic plasticity (Turner et al., 2003; Ring et al., 2007). sAPPα confers antiapoptotic and neuroprotective effects against excitotoxic and oxidative insults and prevents Aβ-induced neurodegeneration in vitro and in vivo (Mattson et al., 1993). Moreover, we have demonstrated that sAPPα can reduce β-secretase activity and Aβ generation (Obregon et al., 2012). Aβ oligomerization is very important in the formation of senile plaques (Roychaudhuri et al., 2009; Yankner and Lu, 2009), so sAPPα should also ameliorate these pathological changes in AD. Recently, Lu et al. (2011) found that baicalein can inhibit Aβ fibrillation and oligomerization, disaggregate preformed Aβ fibrils, and prevent Aβ fibril-induced toxicity in PC12 cells. These results undoubtedly provide several explanations for the beneficial effect of baicalein on AD models.

Our present study also demonstrates that baicalein decreases the production of Aβ and promotes nonamyloidgenic APP processing by activation of GABA_A_ receptor signaling. As shown in Figure 3A,B, baicalein-mediated reduction of Aβ was counteracted by cotreatment with the GABA_A_ receptor antagonist bicuculline. In concert with this finding, baicalein-mediated elevation of sAPPα, and reduction of β-CTF was also inhibited by bicuculline (Fig. 3B–D). GABA is known to be the most important inhibitory neurotransmitter in the CNS, playing a key role in long-term potentiation (Dawson et al., 2006). Previous studies have indicated that activation of GABA_A_ receptor signaling elicits neuroprotection against Aβ-mediated toxicity (Gu et al., 2003; Lee et al., 2005). In addition, elevated GABA signaling appears to have a protective effect on neurogenesis. Tao et al. (2008) found that baicalein was the most potent inhibitor of GABA transaminase, which is the key enzyme metabolizing GABA to its inactive metabolite. Marcade and colleagues have demonstrated a direct relationship among GABA_A_ receptor signaling, α-secretase, and sAPPα, which can directly inhibit the β-secretase activity (Marcade et al., 2008; Obregon et al., 2012).

Taken together, our present study indicates that baicalein can directly reduce Aβ production and promote nonamyloidgenic pathway in vitro and in vivo, thereby improving the learning and spatial memory performance of Tg2576 mice, effects associated with GABA_A_ receptor signaling. Future clinical trials using this compound for treatment of AD are warranted, especially in light of recent vaccine trial failures (Liu et al., 2012).
